# Better objective sleep quality is associated with higher gut microbiota richness in older adults

**DOI:** 10.1007/s11357-025-01524-w

**Published:** 2025-01-31

**Authors:** Maria Teresa Wijaya, Ji-Tseng Fang, Geng-Hao Liu, Yuan-Ming Yeh, Ning-Hung Chen, Chih-Ming Lin, Kuain-Yi Wu, Chih-Mao Huang, Shwu-Hua Lee, Tatia M. C. Lee

**Affiliations:** 1 State Key Laboratory of Brain and Cognitive Sciences, The University of Hong Kong, Hong Kong, Hong Kong; 2 Laboratory of Neuropsychology & Human Neuroscience, The University of Hong Kong, Hong Kong, Hong Kong; 3 Department of Nephrology, Linkou Chang Gung Memorial Hospital, Taoyuan, Taiwan; 4 College of Medicine, Chang Gung University, Taoyuan, Taiwan; 5 Genomic Medicine Core Laboratory, Chang Gung Memorial Hospital, Linkou Taoyuan, 333 Taiwan; 6 Department of Internal Medicine, Linkou Chang Gung Memorial Hospital, Taoyuan, Taiwan; 7 Department of Biological Science and Technology, National Yang Ming Chiao Tung University, Hsinchu, Taiwan; 8 School of Traditional Chinese Medicine, College of Medicine, Chang Gung University, Taoyuan, Taiwan; 9 Division of Acupuncture and Moxibustion, Center for Traditional Chinese Medicine, at Linkou, Chang Gung Memorial Hospital, Taoyuan, Taiwan; 10 Sleep Center, Taoyuan Chang Gung Memorial Hospital, Taoyuan, Taiwan; 11 Sleep Center, Respiratory Therapy, Pulmonary and Critical Care Medicine, at Taoyuan, Chang Gung Memorial Hospital, Taoyuan, Taiwan; 12 School of Traditional Chinese Medicine, Chang Gung University, Taoyuan, Taiwan; 13 Chang Gung Microbiota Therapy Center, Linkou Chang Gung Memorial Hospital, Taoyuan, 333 Taiwan; 14 Graduate Institute of Health Industry Technology, Chang Gung University of Science and Technology, Taoyuan City, 333 Taiwan; 15 Department of Psychiatry, Linkou Chang Gung Memorial Hospital, Taoyuan City, 333 Taiwan; 16 Department of Psychiatry, Chang Gung Memorial Hospital and College of Medicine, Chang Gung University, Taoyuan, Taiwan

**Keywords:** Microbiota, Gut, Sleep, Older adults, Gut-brain axis

## Abstract

Aging is associated with disrupted sleep patterns, such as fragmented sleep and reduced efficiency, leading to negative health outcomes. There is evidence of a bidirectional relationship between sleep and gut microbiota, which plays a key role in the gut-brain axis and overall health. However, studies on this relationship in older adults have limited generalizability and show conflicting results, highlighting the need for further research. This study aimed to investigate the associations between sleep quality and gut microbiota composition in healthy Chinese older adults using subjective and objective sleep measures to capture various aspects of sleep quality and explore potential impacts on emotional well-being and cognitive performance. Subjective sleep quality was assessed using the Pittsburgh Sleep Quality Index (PSQI) questionnaire, while objective sleep quality was measured with actigraphy. Gut microbiota sequencing was performed on stool samples. The results show a robust positive association between gut microbiota richness and objective sleep quality in older adults, independent of subjective sleep quality and demographics, lifestyle, and health covariates. However, no significant link was found between gut microbiota richness and subjective sleep quality. Specific taxa like Bacteroidetes, *Ruminococcus*, *Collinsella*, *Veillonella*, and *Holdemania* were tentatively linked to sleep quality. These findings emphasize the connection between sleep quality and gut microbiota composition in older adults with potential research and clinical implications, improving our understanding of the mechanisms underlying the sleep-gut microbiota relationship and guiding the development of interventions for improving both sleep quality and gut health in older adults.

**Supplementary Information:**

The online version contains supplementary material available at 10.1007/s11357-025-01524-w.

## Introduction

Aging is associated with multiple changes in sleep patterns [1]. Older adults experience more fragmented sleep, increased awakening, reduced sleep efficiency, and shorter total sleep time [2]. Sleep disorders such as insomnia, disordered breathing, and circadian rhythm disturbances are also more commonly found [1]. The decline in sleep quality is associated with an increased risk for a host of negative health outcomes such as systemic inflammation, cardiometabolic disorders, and all-cause mortality [3]. There is accumulating evidence that a bidirectional relationship exists between sleep and gut microbiota composition, which may mediate some of the negative effects associated with poor sleep [4]. Gut microbiota, a collection of microorganisms in the gastrointestinal system, is a key regulator of the gut-brain axis which comprises a communication pathway between the gastrointestinal system and the central nervous system and plays a significant role in human physical and mental health [5–7].

Despite the prevalence of sleep disorder in older adults and the hypothesized critical role of gut microbiota, few studies have investigated the sleep and gut-brain axis relationship in older adults and the results are often conflicting, suggesting the need for more research [8–11]. For example, neither objective nor subjective sleep quality was significantly associated with microbiota diversity measures in one study [10], while another study found that objective sleep efficiency was a significant predictor of microbiota composition [11]. Sleep is a multidimensional construct, including duration, timing, regularity, efficiency, and subjective satisfaction [12]. Furthermore, objective and subjective sleep assessments do not always concur and show distinct associations with health and behavioral outcomes [13–16], highlighting the need to consider both when evaluating sleep quality. However, existing studies in the literature have focused only on very limited aspects of sleep quality measures, such as subjective satisfaction [9], self-report duration [8], timing and regularity [10], and sleep efficiency [11]. These differences in operationalizing sleep quality may explain some of the discrepant findings in the literature.

Additionally, existing studies have limited generalizability as all of them have recruited predominantly Western samples, which were further limited to only men [10], or only older adults with clinical sleep disturbance [11]. Gut microbiota composition is affected by factors such as genetics [17], diet [18], and geography [19]. For example, the healthy Chinese gut microbiota community was predominated by *Bacteroides* and *Faecalibacterium*, while the primary enterotypes in European and American populations were *Bacteroides* and *Firmicutes* [19–21]. These differences make it difficult to generalize the existing literature that is predominated by studies from Western countries. Given the fast growth of the aging population in East Asia due to longer life expectancy [22], it is important to understand the sleep and microbiota gut-brain axis associations in the Chinese population.

The present paper had two objectives. First, we aimed to test the associations between sleep quality and gut microbiota composition in a sample of healthy Chinese older adults. Second, we aimed to comprehensively capture the multiple dimensions of sleep quality by testing both subjective and objective sleep measures. To these ends, we collected sleep questionnaires, actigraphy, and stool samples from community-dwelling adults aged 60 years and older who resided in Taiwan. We performed permutational ANOVA tests for the independent effects of subjective and objective sleep measures on gut microbiota composition, controlling for demographic, health, and lifestyle covariates. Since subjective and objective sleep quality measures have been associated with gut microbiota composition in the literature, we hypothesized that each will have independent positive associations with greater richness and diversity. Additionally, we performed exploratory analysis to probe the associations between sleep quality and gut microbiota composition with emotional well-being and cognitive performance.

## Materials and methods

### Participants

The sample was taken from the Integrating Systematic Data of Geriatric Medicine to Explore the Solution for Health Aging study that aimed to establish a comprehensive database for geriatric medicine for Taiwanese with no major physical or mental disabilities [23]. Community-dwelling adults residing in the Songshan District, Taipei City, Taiwan, or Chang Gung Health and Culture Village, Taoyuan City, Taiwan, were recruited when undergoing health examinations at Chang Gung Memorial Hospital. The inclusion criteria were (1) adults aged 60 years or older; (2) at least 1 visit to Chang Gung Memorial Hospital within 1 year of recruitment; and (3) remained in Taiwan for more than 180 days within 1 year of recruitment. The exclusion criteria were (1) clinical evidence of major organ system abnormalities; (2) history of severe autoimmune disorders; (3) cancer treatment at recruitment; (4) antibiotic treatment within 1 month of recruitment; (5) Ascertain Dementia 8 (AD8) score of 2 or above, mini-mental state examination (MMSE) score of 26 or less; (6) Geriatric Depression Scale score of 5 or above; (7) outpatient follow-up for cognitive problems; (8) physician-diagnosed dementia or major depressive disorder; (9) significant hearing, visual, or cognitive impairments or inability to participate in interviews in a meaningful manner; (10) too frail to stand and walk; (11) sarcopenia. Participants were also excluded if they had 5% weight loss within 1 month or 10% weight loss within half a year, as well as plasma albumin levels that were out of normal range.

The study was approved by the institutional review board of Chang Gung Medical Foundation, Taiwan (approval number 201900702A3). All research procedures adhered to the tenets of the Declaration of Helsinki. Informed consent was obtained from all participants. Participants were given enough time for inquiry or asking the advice of their significant others. To ensure participants’ capacity to consent, a research assistant evaluated their cognitive function by applying the MMSE and AD8 as part of the exclusion criteria described above. Those with cognitive impairment that may hamper their capacity were excluded (MMSE ≦26 or AD8 ≧2). Copyright permission to use the MMSE for study purposes was procured. Participants’ demographics, medical histories, and physical examinations including anthropometric characteristics were recorded. Medical history was obtained through interviews and validated by a review of the medical records.

Table 1 lists the participants’ characteristics. Between September 2019 and October 2020, 128 participants were enrolled. Sixteen enrolled participants did not meet the inclusion criteria. Another 16 participants were excluded due to early withdrawal from the study. Actigraphy data were available for 94 participants. One participant was excluded due to extreme value in sleep efficiency, resulting in 93 participants who were included in the clustering. The average age of participants was 74.19 years (SD = 7.25), comprising 44% males and 56% females. Of these 93 participants, gut microbiota data were available for 42 participants. Only participants with complete data were entered into the analysis, as reflected by the total degrees of freedom (df) of each ANOVA model.

**Table 1:** Participants' characteristics

	*n*	Mean (SD)
Age (years)	93	74.19 (7.25)
Gender		
Male	41	
Female	52	
Education level		
High school or below	29	
Junior college	23	
College or above	41	
Chao1	42	584.94 (131.77)
Shannon	42	4.68 (0.68)
PSQI		
SGS (≤ 5)	47	3.26 (1.17)
SPS (> 5)	46	9.39 (2.89)
Actigraphy		
TST (minutes)	93	396.02 (73.08)
TSTV (minutes)	93	58.22 (27.33)
SE (%)	93	84.24 (5.71)
SOL (minutes)	93	9.31 (5.72)
WASO (minutes)	93	55.46 (25.28)
BT (minutes since 12:00)	93	641.16 (89.96)
Diet		
Ovo-lacto vegetarian	4	
Vegetarian	3	
Omnivore	86	
Exercise level		
None	5	
Below 150 min weekly	19	
150 min or more weekly	69	
BMI	93	23.78 (3.39)
Hypertension		
Yes	46	
No	47	
Sleep disorder		
Yes	20	
No	73	
Hyperlipidemia		
Yes	39	
No	54	
Diabetes mellitus		
Yes	17	
No	76	
Heart disease		
Yes	10	
No	83	
Sleep medication		
Yes	9	
No	83	
N/A	1	
Anti-inflammatory medication		
Yes	1	
No	91	
N/A	1	
Antibiotics medication		
Yes	1	
No	92	
HAM-A	88	4.19 (3.88)
HAM-D	88	2.54 (2.64)
CERAD-NB memory total	87	34.49 (3.63)
ECog	66	6.28 (0.94)

*SD* standard deviation, *PSQI* Pittsburgh Sleep Quality Index, *SGS* subjective good sleepers, *SPS* subjective poor sleepers, *TST* total sleep time, *TSTV* total sleep time variability, *SE* sleep efficiency, *SOL* sleep onset latency, *WASO* wakefulness after sleep onset, *BT* bedtime, *BMI* body mass index, *HAM-A* Hamilton rating scale for anxiety, *HAM-D* Hamilton rating scale for depression, *CERAD-NB* Consortium to Establish a Registry for Alzheimer’s Disease Neuropsychological Battery, *ECog* everyday cognition

### Questionnaires

#### Pittsburgh Sleep Quality Index (PSQI)

A Chinese translation of the PSQI, a 19-item self-report questionnaire was used to assess perceived sleep quality over a one-month interval [24, 25]. The questionnaire evaluates seven dimensions of sleep quality, including subjective sleep quality, sleep latency, duration, habitual sleep efficiency, sleep disturbances, use of sleeping medication, and daytime dysfunction. Each component was scored on a scale from 0 (best) to 3 (worst). The component sub-scores were added to yield a global score ranging from 0 to 21, with higher scores indicating poorer sleep quality. Following other works [24, 26–28], a threshold of 5 was used to classify participants as “subjective good sleepers” (SGS, PSQI ≤ 5) or “subjective poor sleepers” (SPS, PSQI >5).

#### Hamilton rating scale for depression (HAM-D) and anxiety (HAM-A)

A Chinese translation of the 17-item HAM-D was used to measure the severity of depression in the participants [29, 30]. A Chinese translation of the 14-item HAM-A was used to measure anxiety symptoms [31]. A trained research assistant conducted a face-to-face interview and scored the severity. The HAM-D has been used widely in psychological and neuropsychiatric studies as an indicator of depressive symptoms and severity of depression, and it has very high interrater reliability on both single items and the total score [32].

#### Everyday cognition (ECog)

Participants completed the 39-item ECog questionnaire [33], covering six domains: everyday memory, language, visuospatial and perceptual abilities, planning, organization, and divided attention. The score for each item was based on a 4-point scale: 1 = better or no change compared to 10 years earlier; 2 = questionable/occasionally worse; 3 = consistently a little worse; 4 = consistently much worse. An option to respond “I don’t know” was also included. Responses to all 39 items were summed to calculate the total score, excluding the “I don’t know” responses. The average scores were adjusted for the number of excluded responses.

### Cognitive tests

#### Consortium to Establish a Registry for Alzheimer’s Disease Neuropsychological Battery (CERAD-NB)

CERAD-NB [34] has been extensively used to evaluate cognitive decline and dementia, comprising five subsets: an executive domain of category verbal fluency test (maximum score = 24), a language domain of the modified Boston Naming Test (maximum score = 15), a memory domain of the word-list-learning test (maximum score = 30) with delayed recall (maximum score = 10) and recognition (maximum score = 10), a visuospatial domain of the constructional praxis test (maximum score = 11), and constructional recall test (maximum score = 11). A memory total score was calculated by adding the word-list-learning test, delayed recall, and recognition scores (maximum score = 50) [23].

### Actigraphy data collection and pre-processing

Participants used the Actiwatch2 accelerometer (Philips Respironics) for objective sleep assessment. The accelerometer was worn on the non-dominant wrist for up to 14 consecutive days. To ensure compliance during this period, participants were specifically reminded to continuously wear the device on their non-dominant hands and not to remove it. Additionally, a dedicated research assistant inquired about the previous night’s sleep as part of a brief sleep diary and to confirm the wearing status. After the 2-week recording period, the assistant collected the devices for data extraction and preliminary analysis using a computer. The brief sleep diary and preliminary actigraphy data were then handed over to a sleep specialist for the final interpretation of wake-sleep patterns and activity levels.

During analysis, a sleep technician initially determined sleep and wake periods using the Philips Actiware software. When the total activity count was less than or equal to the wake threshold value, the period was defined as sleep; when the total activity count was greater than the wake threshold value, the period was considered awake. Subsequently, a sleep specialist reviewed the actigraphy data, incorporating activity and light exposure records along with the participants’ sleep diaries to finalize the actigraphy readings.

Sleep parameters included average bedtime (BT; the time a participant got into bed converted into a continuous variable in minutes). Sleep duration and its variability were computed as the average (TST) and standard deviation (TSTV) of total sleep time (the difference between sleep onset and offset). Sleep efficiency (SE) was estimated as the average percentage of time spent sleeping while in bed. Sleep disturbance was estimated using the average of sleep onset latency (SOL; the number of minutes it took a participant to fall asleep) and average of wakefulness after sleep onset (WASO; the number of minutes awake between sleep onset and the end of the sleep period).

### Stool sample collection

Stool samples were collected using the Longsee Fecalpro Kit (Longsee Medical Technology Co., China), equipped with a preservation solution. Under the guidance of health educators, participants collected stool samples at home. The samples were returned to the laboratory on the same day, where researchers processed and aliquoted them before storing them at −80°C to ensure the preservation of sample quality.

### Gut microbiota sequencing and analysis

The genomic DNA was extracted from stool samples using the QIAamp PowerFecal Pro DNA Kit (Qiagen, Germany). The extracted DNA underwent amplicon sequencing, targeting the 16S rRNA V3-V4 region [35]. PCR products were purified and subjected to index PCR prior to sequencing with a MiSeq System using the MiSeq Reagent Kit v3 (600 cycles). The paired-end reads obtained were demultiplexed using MiSeq Reporter v2.6. Subsequent bioinformatics analysis, including merging, filtering, and clustering of reads into zero-radius operational taxonomic units (zOTUs), was performed using tools such as USEARCH (v11.0.667_i86linux64) (https://drive5.com/usearch), the Cutadapt v3.4 package [36], and UNOISE3 [37]. Taxonomic assignments were conducted using the SINTAX algorithm [38] with an RDP (v18) reference species database. Additionally, alpha (within-sample) diversity metrics were estimated. Richness, indicating the number of distinct species found in the gut, was measured using the Chao1 richness index [39]. Diversity, which considers both microbiome richness and the evenness of species abundance, was measured using Shannon’s diversity index [40].

### Data analysis

#### Clustering of actigraphy data

Because there is currently no well-established standard to classify sleep quality based on actigraphy variables, we opted for a data-driven approach using clustering. Six actigraphy-derived variables (BT, TST, TSTV, SE, SOL, and WASO) were considered as features to develop objective sleep clusters, encompassing multiple dimensions of sleep [12]. Outliers were defined as values equal to or greater than 6 SD above or below the means.

Cluster analysis was performed using R version 4.2.2 [41]. All variables were standardized prior to analysis. A *k*-means clustering algorithm [42] with 25 initial configurations was used to determine specific objective sleep clusters. The optimal number of clusters was identified by silhouette analysis [43] and by considering the interpretability of the clusters. To test the influence of algorithm choice on the results, clustering was repeated using a spectral clustering algorithm [44] which does not make assumptions about cluster shapes. The following R packages were used: Spectrum (v1.1), clusterSim (v0.51-3), and factoextra (v1.0.7).

#### Covariates

Demographic, lifestyle, and health characteristics were considered as covariates. Demographic characteristics consisted of age, sex, and education level, which was entered as a categorical covariate with three levels (high school graduate or lower, junior college, and college or above). Lifestyle covariates consisted of exercise level and diet type, both entered as categorical variables with three levels (exercise level: 150 min or more weekly, below 150 min weekly, none; diet type: lacto-ovo vegetarian, vegetarian, and omnivore). Health covariates consisted of body mass index (BMI), clinical conditions, and medication intake. Hypertension, sleep disorder, hyperlipidemia, diabetes mellitus, heart diseases, as well as intake of sleep medications and anti-inflammatory medications were each entered as a categorical variable with two levels (1 = yes, 0 = no). Antibiotic intake was also included as a health covariate (1 = yes, 0 = no) to account for one participant who reported incidental intake despite the study’s exclusion criteria.

#### Statistical analysis

The model for each of the alpha diversity outcomes was tested using permutation-based ANOVA in R package lmPerm (v2.1.0). Number of permutations was set to 10,000 for all tests. The effects of objective and subjective sleep quality were evaluated simultaneously to assess their main effects. Objective sleep quality was entered as a categorical variable according to the actigraphy data clusters as described in the “Results” section. Subjective sleep quality was entered as a categorical variable with two levels (SGS, SPS). Additional covariates were included in each successive model. Model 1 included the covariates age, sex, and education level; model 2 added diet and exercise level; model 3 additionally included BMI and clinical conditions (hypertension, sleep disorder, hyperlipidemia, diabetes mellitus, heart diseases); model 4 added sleep medication, anti-inflammatory medication, and antibiotics intake.

Additional permutation-based ANOVAs were performed to explore whether the abundance of any taxon was associated with objective and subjective sleep quality. Taxa that had more than 30% zeros were removed to reduce multiple testing and the effect of zero inflation. Exploratory analyses were also conducted to probe the associations between sleep quality and gut microbiota composition with emotional well-being and cognitive performance. HAM-A, HAM-D, CERAD-NB memory total, and ECog scores were entered as dependent variable in separate models. Sleep quality measures and gut microbiota indices that showed significant effects from the main analysis were entered as independent variable separately. The Bonferroni procedure was used to correct for multiple comparisons. The first model with age, sex, and education level as covariates was tested. Further covariates were added only if the first model was significant.

## Results

### Actigraphy-derived sleep clusters

Six actigraphy-derived variables were considered to develop sleep clusters; however, two were dropped. BT was dropped because its inclusion resulted in a greatly imbalanced cluster size (*k* = 2 with *n* = 85 and 8, respectively) while WASO was dropped because it was highly correlated with sleep efficiency (Pearson’s *r* = −.88). The absolute values of pairwise correlations between the remaining four variables were all below .4.

**Fig. 1:**
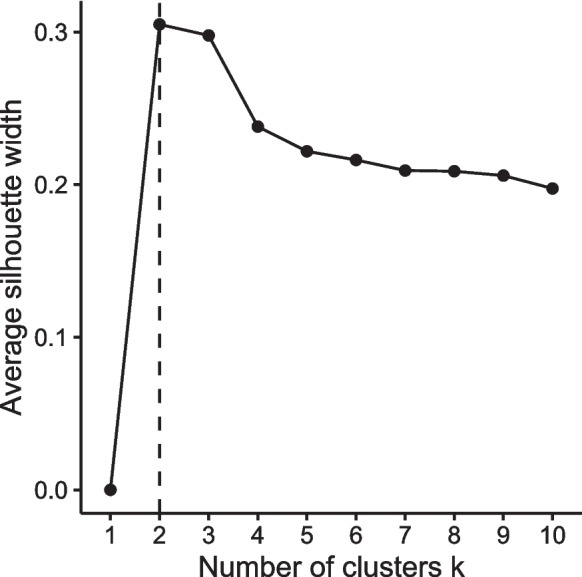
Silhouette plot showing the average silhouette width over values of k = 1 to k = 10

Silhouette analysis suggested that dividing participants into two groups was most appropriate. Choosing *k* = 2 and *k* = 3 resulted in average silhouette widths with negligible difference (Fig. 1); however, we chose *k* = 2 as the optimal number of clusters due to considerations of cluster sizes and their interpretability (see Supplementary Figure 1). Table 2 summarizes the actigraphy characteristics by cluster and *p*-values from one-way permutation-based ANOVA with objective sleep cluster as the only factor. The two clusters significantly differed in all four actigraphy-derived sleep variables (all *p*s < .05). Based on these characterizations, cluster 1 was named “objective poor sleepers” (OPS) and cluster 2 was named “objective good sleepers” (OGS). OPS (30%; *n* = 28) was characterized by longer but inefficient sleep with higher day-to-day variability. Compared to OGS (70%; *n* = 65), OPS displayed significantly longer sleep duration, lower sleep efficiency, higher sleep onset latency, and higher variability in total sleep time. The greatest effect size was observed for sleep onset latency (*η*_*p*_^2^ = .58). We also tested whether OPS and OGS significantly differed in their PSQI scores and the result was non-significant (*p* = .19; OPS mean = 6.86 SD = 3.72; OGS mean = 6.05 SD = 3.80; *η*_*p*_^2^ = .01), in line with the view that subjective and objective sleep measures provide unique insights into sleep quality [15].

**Table 2:** Actigraphy characteristics in all participants by cluster (*k*-means)

	Cluster 1 (*n* = 28) Mean (SD)	Cluster 2 (*n* = 65) Mean (SD)	*p*-value (*η*_*p*_^2^)
TST (minutes)	440.43 (92.35)	376.89 (53.34)	< .001* (.16)
SE (%)	82.50 (6.30)	84.98 (5.32)	.04* (.04)
SOL (minutes)	15.89 (5.09)	6.48 (3.00)	< .001* (.58)
TSTV (minutes)	71.61 (37.10)	52.46 (19.52)	< .001* (.10)

*P*-values (and the corresponding effect size in brackets) indicate results from one-way permutation-based ANOVAs with actigraphy cluster as a factor with two levels. Asterisk (*) indicates a statistically significant effect at *p* < .05. Total df for all models = 92

*TST* total sleep time, *SE* sleep efficiency, *SOL* sleep onset latency, *TSTV* total sleep time variability

### Associations between sleep and gut microbiota alpha diversity

**Fig. 2:**
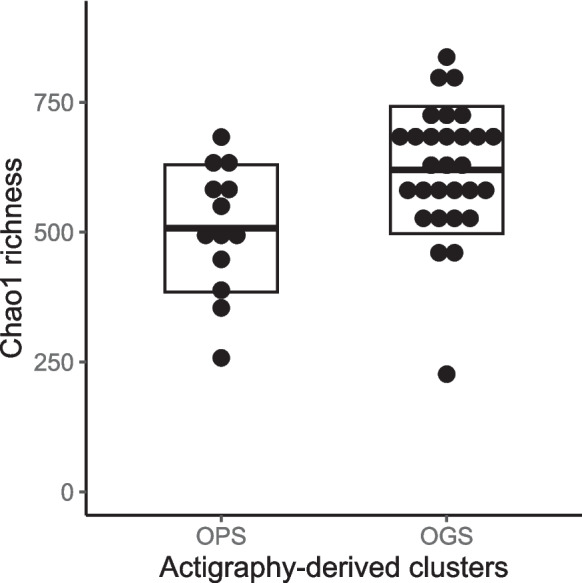
Graphical illustration of the statistically significant difference in Chao1 richness according to actigraphy-derived objective sleep measures. Participants were divided into two clusters based on k-means clustering. Dots represent individual data points. Crossbars represent group means ± SD. The difference was statistically significant at *p* < .05 (Table 3). OPS, objective poor sleepers; OGS, objective good sleepers

To assess whether objective and subjective sleep quality were associated with overall gut microbiota richness and diversity, we used permutation-based ANOVAs with Chao1 richness and Shannon’s diversity as a dependent variable. Actigraphy-derived clusters and PSQI groups were entered as factors with two levels each. We progressively adjusted for additional covariates to control for potential confounders. Across all models, objective sleep quality was associated with Chao1 richness (Tables 3 and 4). For example, in the fully adjusted model, OGS and OPS significantly differed in Chao1 richness with a large effect size (*p* = .02, *η*_*p*_^2^ = .20). In line with our hypothesis, OGS had a significantly higher microbiota richness when compared to OPS (Fig. 2). OGS and OPS also significantly differed in Shannon’s diversity index but the effect became non-significant in the models which included additional lifestyle and health covariates. PSQI groups did not significantly differ in either Chao1 richness or Shannon’s diversity.

**Table 3:** Permutation-based ANOVAs testing the associations between sleep and Chao1 index

	Model 1	Model 2	Model 3	Model 4
Actigraphy^a^ (df = 1)	.008* (.17)	.03* (.14)	.03* (.16)	.02* (.20)
PSQI^b^ (df = 1)	.18 (.04)	.18 (.05)	.30 (.06)	.40 (.02)

Cells show the *p*-values and the corresponding effect size computed as *η*_*p*_^2^ in brackets. Asterisk (*) indicates a statistically significant effect at *p* < .05. Total df for each model = 41. Model 1 included the covariates age, sex, and education level; model 2 added diet and exercise level; model 3 additionally included BMI and clinical conditions (hypertension, sleep disorder, hyperlipidemia, diabetes mellitus, heart diseases); model 4 added sleep medication, anti-inflammatory medication, and antibiotics intake

^a^Participants were divided into two actigraphy-derived clusters based on *k*-means clustering

^b^Participants were divided into two PSQI-based groups according to a total score cut-off of 5

**Table 4:** Permutation-based ANOVAs testing the associations between sleep and Shannon index

	Model 1	Model 2	Model 3	Model 4
Actigraphy^a^ (df = 1)	.02* (.15)	.06 (.11)	.56 (.09)	.08 (.13)
PSQI^b^ (df = 1)	.27 (.03)	.18 (.04)	.18 (.06)	.34 (.03)

Cells show the *p*-values and the corresponding effect size computed as *η*_*p*_^2^ in brackets. Asterisk (*) indicates a statistically significant effect at *p* < .05. Total df for each model = 41. Model 1 included the covariates age, sex, and education level; model 2 added diet and exercise level; model 3 additionally included BMI and clinical conditions (hypertension, sleep disorder, hyperlipidemia, diabetes mellitus, heart diseases); model 4 added sleep medication, anti-inflammatory medication, and antibiotics intake

^a^Participants were divided into two actigraphy-derived clusters based on *k*-means clustering

^b^Participants were divided into two PSQI-based groups according to a total score cut-off of 5

To test the influence of algorithm choice on the results, clustering was repeated using a spectral clustering algorithm. Participants were divided into two clusters according to four actigraphy-derived variables (TST, TSTV, SE, and SOL). The spectral clustering algorithm resulted in two clusters that significantly differed in SE and SOL but did not significantly differ in TST and TSTV (Supplementary Table 1). The first cluster had indicators of better objective sleep quality (higher SE and lower SOL) and was designated “sOGS” (objective good sleeper based on spectral clustering). The other cluster was designated “sOPS” (objective poor sleepers based on spectral clustering). Similar to the results for *k*-means, the two clusters did not significantly differ in PSQI total scores, indicating similar levels of subjective sleep quality (Supplementary Table 1). Most importantly, results from permutation-based ANOVA with spectral clustering-based assignment as a factor (two levels; sOGS and sOPS) led to the same conclusion regarding the relationship between Chao1 richness and objective sleep quality. In the fully adjusted model, sOGS and sOPS significantly differed in Chao1 richness (*p* = .01, *η*_*p*_^2^ = .19) (Supplementary Table 2). Better objective sleep quality was associated with significantly higher microbiota richness (Supplementary Figure 2).

### Associations between sleep and gut microbiota composition at phylum and genus level

We explored whether the abundance of any taxa differed by sleep quality groups. The first model with age, sex, and education level was tested using permutation-based ANOVAs at the phylum and genus levels. Bonferroni correction was applied to control for false positives, resulting in a threshold for significance at *p* < .01 for the four tests conducted at the phylum level and at *p* < .001 for the 42 tests at the genus level.

Five associations were statistically significant at the uncorrected threshold (Supplementary Tables 3–4). Participants with good and poor objective sleep quality differed in the abundance of Bacteroidetes (*p* = .02, *η*_*p*_^2^ = .13), *Ruminococcus* (*p* = .02, *η*_*p*_^2^ = .12) and *Veillonella* (*p* = .004, *η*_*p*_^2^ = .19). Better objective sleep quality was associated with lower abundance of Bacteroidetes (OPS mean = 46.93; SD = 13.83; OGS mean = 36.82; SD = 12.39) and *Veillonella* (OPS mean = 1.40; SD = 1.79; OGS mean = .30; SD = .66). On the other hand, better objective sleep quality was associated with higher abundance of *Ruminococcus* (OPS mean = 1.34; SD = 2.00; OGS mean = 3.59 SD = 4.81). Participants with good and poor subjective sleep quality differed in the abundance of *Collinsella* (*p* = .03, *η*_*p*_^2^ = .14) and *Holdemania* (*p* = .04, *η*_*p*_^2^ = .09). Better subjective sleep quality was associated with lower abundance of *Collinsella* (SPS mean = .29; SD = .35; SGS mean = .14; SD = .18) and *Holdemania* (SPS mean = .02; SD = .03; SGS mean = .01; SD = .02). However, none of these findings survived the multiple comparison corrections.

### Associations between sleep and gut microbiota with cognitive performance and emotional well-being

Finally, we explored whether objective sleep quality or gut microbiota richness were associated with measures of cognitive performance and emotional well-being. Cognitive performance was measured by ECog total scores as well as the memory total scores from CERAD-NB. Emotional well-being was measured by total HAM-A and HAM-D scores. Following our findings described earlier on the associations between microbiota richness and objective sleep quality, only Chao1 richness and *k*-means-based actigraphy-derived cluster were considered in this analysis. In one set of five models, the actigraphy-derived sleep profile was entered as the independent variable. In another set, Chao1 richness index was entered as the independent variable. The threshold for significance was determined to be *p* < .01 for each set. Age, sex, and education level were included as covariates using permutation-based ANOVAs. Before multiple comparison corrections, only objective sleep quality was marginally associated with CERAD-NB memory total scores (*p* = .05, *η*_*p*_^2^ = .05). No other significant result was found (Supplementary Tables 5–6).

## Discussion

This study tested the associations between sleep quality and gut microbiota composition in older adults. We considered both subjective and objective sleep quality. Subjective sleep quality was measured using PSQI, a validated self-report questionnaire that measures sleep quality and disturbances retrospectively over a 1-month period. Objective sleep quality was measured using actigraphy, which is not subject to participants’ self-report bias and can be useful in assessing sleep variables about which participants lack insight. Clustering was used to classify individuals with good and poor objective sleep quality, allowing a data-driven consideration of multiple actigraphy variables simultaneously. This study extends the literature on the microbiota-gut-brain axis in older adults in several ways. We employed both objective and subjective measures of sleep quality in a group of healthy older adults from the Chinese population, which is still underrepresented in the literature. Our results complement those from previous studies with older adult participants from predominantly Western countries, which employed only limited measures of sleep quality [8, 9], recruited only men [10], or recruited only older adults with clinical sleep disturbance [11].

In line with our hypothesis, we found that individuals with better objective sleep quality had richer gut microbiota. We also found a significant association between objective sleep quality and gut microbiota diversity; however, the result was no longer significant after controlling for lifestyle and health covariates. In contrast to our hypothesis, we did not find any significant associations between subjective sleep quality with either richness or diversity. Two exploratory analyses were conducted. The first was to test the association between objective and subjective sleep quality with microbiota taxa abundance. We found *Ruminococcus* abundance as an indicator of good objective sleep quality and Bacteroidetes, *Veillonella*, *Collinsella*, and *Holdemania* abundance as indicators of poor sleep quality. However, these taxa-level effects were not significant after multiple comparison corrections. The second exploratory analysis tested the associations between objective sleep quality and microbiota richness with cognitive performance and emotional well-being, none of which was statistically significant.

Our main finding is that individuals with better objective sleep quality (i.e., shorter sleep duration, higher sleep efficiency, shorter sleep latency, and low sleep duration variability) had richer gut microbiota. The association was independent of the effect of subjective sleep quality and remained significant even after controlling for the effect of age, sex, education, lifestyle, medication intake, and health status. Microbiota diversity also differed between good and poor objective sleep quality groups; however, the difference became statistically non-significant when diet, exercise level, and other lifestyle and health covariates were added. These results show that when compared to individuals with poor objective sleep quality, those with good objective sleep quality had a greater number of distinct gut microbiota species. They may also differ in the evenness of the species abundance distribution, but this may partially be explained by variability in their diet and exercise levels rather than their objective sleep quality per se.

Contrary to our finding, a previous study on older adults that employed objective sleep quality measures did not find any significant association with richness [10]. The authors used two variables derived from actigraphy, which measured the timing of peak activity in a 24-hour cycle and its regularity. The four variables we considered for clustering analysis did not include any measure of timing regularity; however, we considered total sleep time variability which is the day-to-day regularity of sleep duration. Our supplementary spectral clustering analysis produced two groups that did not differ in their total sleep time variability but still significantly differed in Chao1 richness. We speculated that sleep regularity, either in timing or sleep duration, may be less relevant for the association between gut microbiota richness and objective sleep quality.

On the other hand, a study of 720 healthy adults found that greater actigraphy-measured sleep duration variability, greater WASO, and lower sleep efficiency were associated with lower richness [45]. Another study of 26 young adult males found that richness was positively associated with actigraphy-based sleep efficiency. Richness was also positively correlated with total sleep time and negatively associated with WASO; although, these were not statistically significant [46]. Despite some differences in actigraphy variables, overall, these two studies and our results show that positive sleep measures (e.g., greater sleep efficiency) were associated with richer gut microbiota while adverse sleep measures (e.g., greater sleep onset latency) were associated with less rich microbiota. Greater gut microbiota richness is thought to confer beneficial effects, for example, higher Chao1 is linked to better metabolic health and better performance in a reaction time task [47, 48].

We did not find any significant effect of subjective sleep quality on richness and diversity measures. Prior studies on older adults have used varying measures of subjective sleep quality, such as PSQI [9, 10], self-report sleep duration [8], and self-report insomnia symptoms [11]. Consistent with our finding, a large study of 606 men with an average age of 84 years found no significant difference in alpha diversity, as measured using Faith’s phylogenetic diversity, between men with poor and good subjective sleep quality, as measured using PSQI [10]. Similarly, self-report sleep duration and self-report insomnia symptoms were not associated with measures of alpha diversity [8, 11]. However, in young and middle-aged adults, the majority of studies reported significant associations between PSQI scores and other subjective sleep measures with gut microbiota indices ([8, 9, 45, 49–52]; but see [53]).

Our result suggests that in older adults, subjective measures of sleep quality do not explain additional variance beyond what is captured by objective sleep quality measures. Taken together with the existing literature, this suggests that the relationship between sleep quality and gut microbiota composition may best be captured by objective sleep measures. It is currently unclear why subjective and objective sleep quality is differently associated with gut microbiota alpha diversity. Nevertheless, the dissociation is in line with the view that these two measures target different aspects of sleep in older adults [15, 54]. Inaccurate judgment of sleep quality due to age-related cognitive decline is an unlikely explanation as the discrepancy between subjective and objective sleep measures in older adults is unrelated to cognitive status [15]. In some cases, older adults may overestimate their sleep quality because the deterioration in their sleep quality happens gradually and they adjust their expectation of normal sleep quality over time [26, 54].

We found that good objective sleep quality was associated with a lower abundance of Bacteroidetes. This phylum comprises approximately 7000 different species of gram-negative bacteria. Although Bacteroidetes ferment polysaccharides to produce metabolites such as short-chain fatty acids that are associated with reduced inflammation and other beneficial effects [55], they have also been implicated in age-related inflammatory neurodegeneration [56]. Several existing studies in mice and humans have suggested a bidirectional relationship between sleep quality and Bacteroidetes abundance [46, 50, 57–61]. However, the results were often conflicting, for example, sleep fragmentation in mice was linked to both increased and decreased Bacteroidetes abundance in two separate studies [57, 58]. Nevertheless, our finding is largely in line with prior findings on older adults which reported a predominance of Bacteroidetes in the gut microbiota of those with sleep disorders [11].

In contrast, better objective sleep quality was associated with more abundant *Ruminococcus*, a genus of gram-positive microbes and a member of the Firmicutes phylum. In non-human animals, paradoxical sleep deprivation led to dysbiosis which featured increased *Ruminococcus* abundance [62]. In humans, *Ruminococcus* is associated with an increased risk for obstructive sleep apnoea [63]. However, in line with our findings, a study of healthy young adults found that an increased abundance of *Ruminococcus* was associated with better sleep quality [50] and consumption of a sleep-enhancing supplement [64]. In older adults, greater abundance of *Ruminococcus* sp. was associated with better objective sleep regularity [10].

Additionally, we found that good objective sleep quality was associated with a lower abundance of *Veillonella* while good subjective quality was associated with a lower abundance of *Collinsella* and *Holdemania*. *Veillonella* and *Holdemania* are both members of the Firmicutes. Unlike *Holdemania* and most other members of the Firmicutes phylum that are gram-positive, *Veillonella* are gram-negative bacteria. *Collinsella* are gram-positive bacteria belonging to the phylum Actinomycetota. Our finding on *Collinsella* is largely consistent with the existing literature, showing a negative association with sleep quality. *Collinsella* is enriched in insomnia patients [65], random eye movement (REM) sleep behavior disorder [66], and was positively associated with daytime sleepiness [60]. However, the literature is less consistent for the other two genera. Two studies found conflicting results on *Veillonella*. In one study, subjective poor sleepers showed significant enrichment of *Veillonella* compared to subjective good sleepers [67]; however, in another study, *Veillonella* was among the predominant genera in individuals with normal sleep when compared to those with sleep disorder [61]. Similarly, contradictory findings exist for *Holdemania*. The genus was negatively associated with adverse sleep measures, including the number of awakenings [46] and risk for obstructive sleep apnoea [68]. However, consumption of a sleep-enhancing supplement was found to decrease *Holdemania* abundance [64].

Clearly, the literature is still mixed with regard to the relationships between specific bacterial taxa and sleep quality, even in the relatively few studies that recruited only older adults [8, 9, 11]. These studies have highlighted different taxa, including the genus *Lachnoclostridium* [11], the phyla Verrucomicrobia and Lentisphaerae [9], and the genera *Sutterella* and *Pseudomonas* [8]. Our findings may indicate that *Ruminococcus* has a beneficial relationship with sleep quality while Bacteroidetes, *Veillonella*, *Collinsella*, and *Holdemania* have adverse relationships. However, given the inconsistent findings in the literature and the risk of false positives, this conclusion must be taken cautiously and awaits future research efforts.

The relationship between sleep and gut microbiota is likely bidirectional. Sleep is an extended period of fasting and sleep disturbances may influence daily feeding/fasting cycles, which affect the diurnal oscillations of the gut microbiota [69]. Diurnal rhythmicity in mice exposes the intestinal epithelium to varying bacterial species and their metabolites over a day with consequences on the host physiology, disease vulnerability, as well as transcriptional, epigenetic, and metabolite oscillations [70].

The detrimental effects of gram-negative bacteria such as Bacteroidetes spp. and *Veillonella* spp. may be linked to the release of lipopolysaccharide (LPS) endotoxins, particularly at high doses [71–74]. LPS endotoxins are components of the cell wall that are released when bacteria are destroyed or during cell division. Lipoteichoic acid, a major component of the gram-positive bacterial cell wall, had similar effects on sleep architecture in mice; although, it is less well-studied [75]. In humans, a high dose of intravenous LPS endotoxins administration elicited a strong host defense activation and disrupted sleep quality, doubling the amount of wakefulness and decreasing the amount of non-REM sleep during the first half of the night followed by a rebound in the second half [76]. A similar dose-dependent effect has been found in non-human animals [73, 77, 78]. The pro-inflammatory effects of LPS endotoxins may be more severe in the elderly as the gastrointestinal tract and blood-brain barriers tend to increase their permeability due to aging [79].

The beneficial effects of *Ruminococcus* on human health have been linked to the production of butyrate, a short-chain fatty acid [80]. Oral and intraportal administration of butyrate in mice and rats elicited an increase in non-REM sleep and suppressed REM sleep [75]. Butyrate is produced by live bacteria in the intestine and is anti-inflammatory, mediating transcriptional inhibition of cytokines and inflammatory proteins [50]. Therefore, although butyrate’s effect is similar to the changes in sleep architecture induced by LPS endotoxins, the effects induced by butyrate are not linked to inflammatory processes.

Admittedly, our analyses were limited to the phylum and genus levels and cannot speak directly to these mechanistic explanations so our discussion must remain speculative. Besides *Ruminococcus*, other members of Firmicutes including *Collinsella* and *Veillonella* as well as certain members of the phylum Bacteroidetes can also produce butyrate [81]. There are also other mechanisms for a gut microbiota-sleep relationship such as via the conversion of primary to secondary bile acids by the gut microbiota and via the regulation of the sleep-wake cycle by serotonin, which is largely derived from dietary tryptophan that is absorbed in the gut [82].

This study has several limitations. First, our sample size was on the lower end and may not fully represent the Chinese older adult population. We have attempted to partially address the issue of low sample size by using non-parametric statistics with resampling to improve the precision of our significance testing. The low sample size for the tests on the associations between gut microbiota with cognitive performance and emotional well-being may explain the lack of significant findings, contrary to others [83]. Second, our cross-sectional data precludes us from making any definitive conclusion on the direction of the effect between objective sleep and gut microbiota. Future studies should replicate our findings in a larger sample of Chinese or other East Asian older adults. Third, analysis of sleep stages using polysomnography may provide additional insight into how gut microbiota is related to sleep architecture in older adults [66, 75, 76], complementing measures of sleep quality obtained from actigraphy and self-report that we utilized in this study.

## Conclusions

Gut microbiota is a significant component of the gut-brain axis with important influences on physical and mental health. Our study found a robust positive association between gut microbiota richness and objective sleep quality, after accounting for subjective sleep quality, and important lifestyle, demographic, and health covariates. On the other hand, we found no significant association between gut microbiota richness and subjective sleep quality. Our results also tentatively identified several taxa as being linked to sleep quality, including Bacteroidetes, *Ruminococcus*, *Collinsella*, *Veillonella*, and *Holdemania*.

Overall, our findings strengthen the link between sleep quality and gut microbiota composition in older adults. Several important implications can be drawn. First, objective sleep assessment, which can be done at home using actigraphy, may serve as an important correlate of gut health in older adults. While it may be more logistically taxing, future studies investigating the association between sleep quality and gut microbiota composition may consider incorporating objective sleep measures. Second, we identified two potentially important genera with low abundance in our sample (*Collinsella* and *Holdemania*). Low-abundance bacteria are typically excluded from analysis; however, their important roles in maintaining gut health have recently started to be recognized [83]. Lastly, in the long term, our findings may contribute to a better understanding of the mechanisms underpinning the relationship between sleep and microbiota gut-brain axis, and shape the search for effective interventions. Future studies could explore more fine-grained taxa-level analysis based on the groups identified in our study and others. Sleep disturbances are common in older adults and our study suggests that interventions aimed to improve objective sleep quality may have wide-ranging impacts that include improvement in gut health. Conversely, supplements to increase microbiota richness or abundance of specific groups may help improve sleep quality.

## Supplementary Information

Below is the link to the electronic supplementary material.

Supplementary file1 (DOCX 238 KB)

## Data Availability

The processed data and code utilized in this study can be made available upon a reasonable request. For any requests or needs regarding the data and code, we encourage early communication with the corresponding author(s) to obtain the necessary permissions for collaboration. The raw data are not publicly available due to a lack of informed consent from the participants and ethical approval for public data sharing.
