# Supplementary material for: Better objective sleep quality is associated with higher gut microbiota richness in older adults
Source: GeroScience. 2025 Jan 31;47(3):4121–37. doi: 10.1007/s11357-025-01524-w (PMC12181545; doi:10.1007/s11357-025-01524-w)
Supplement: Supplementary file 1 — Supplementary file1 (DOCX 238 KB) [file 11357_2025_1524_MOESM1_ESM.docx]

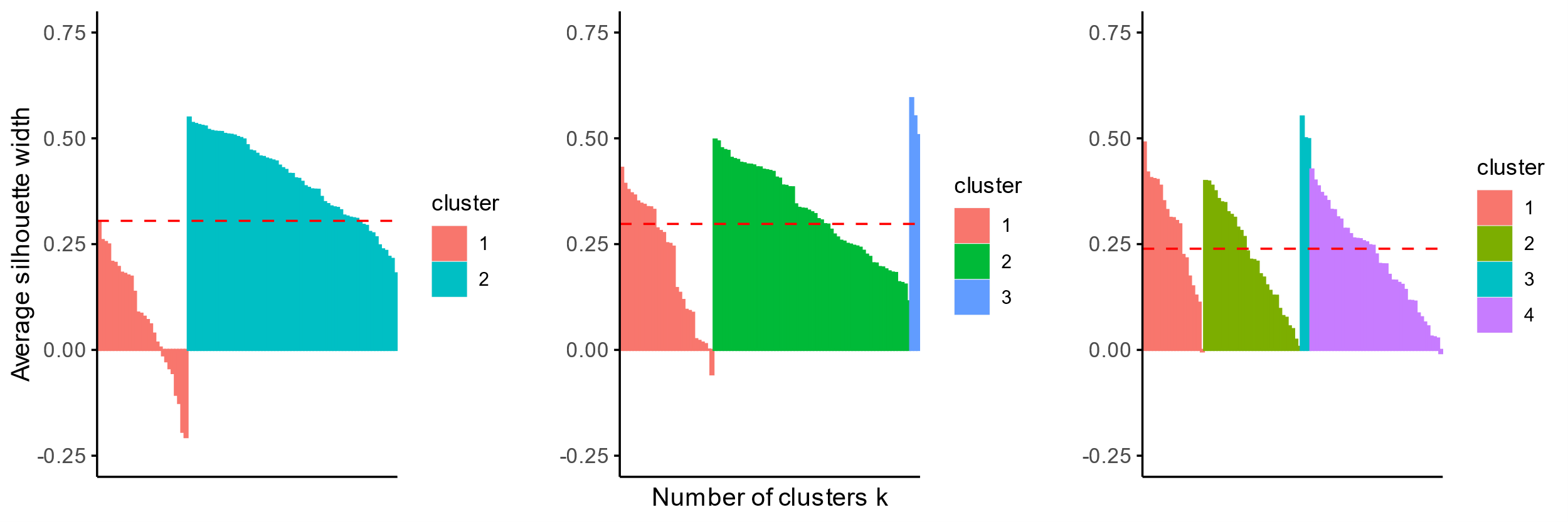


**Supplementary Fig. 1** Silhouette plots showing the silhouette width of each sample in the clusters. Dotted lines indicate the average silhouette width. The width reflects how close each point in a cluster is to the points in the neighbouring clusters. Higher value indicates better separation. The optimal number of clusters k was chosen primarily by maximizing the average and individual silhouette widths over a range of possible k values as well as by considering the interpretability of the solution. The plots show that choosing k = 2 (left) and k = 3 (middle) resulted in similar average silhouette widths (.305 and .298 respectively). The former had more points with negative silhouette width, however the cluster size in the latter was greatly imbalanced with only 3 data points in the third cluster. Therefore, we chose k = 2 as the number of optimal clusters

**Supplementary Table 1** Characteristics in all participants by cluster (spectral clustering)

|  | **Cluster 1 (n = 72)**  **Mean (SD)** | **Cluster 2 (n = 21)**  **Mean (SD)** | ***p*-value**  **(η_p_^2^)** |
| --- | --- | --- | --- |
| TST (minutes) | 396.19 (78.08) | 395.43 (54.10) | 1.00 (<.001) |
| SE (%) | 85.46 (5.29) | 80.05 (5.19) | <.001* (.16) |
| SOL (minutes) | 6.79 (3.09) | 17.95 (3.93) | <.001* (.67) |
| TSTV (minutes) | 58.30 (29.29) | 57.95 (19.74) | .96 (<.001) |
| PSQI | 6.35 (3.92) | 6.10 (3.32) | .69 (.001) |

*P*-values (and the corresponding effect size in brackets) indicate results from one-way permutation-based ANOVAs with actigraphy cluster as a factor with two levels. Asterisk (*) indicates statistically significant effect at *p* < .05. Total df for all models = 92. TST = Total Sleep Time, SE = Sleep Efficiency, SOL = Sleep Onset Latency, TSTV = Total Sleep Time Variability, PSQI = Pittsburgh Sleep Quality Index

**Supplementary Table 2** Permutation-based ANOVAs testing the associations between sleep and Chao1 index

|  | **Model 1** | **Model 2** | **Model 3** | **Model 4** |
| --- | --- | --- | --- | --- |
| Actigraphy^a^ (df = 1) | .01* (.15) | .04* (.12) | .01* (.18) | .01* (.19) |
| PSQI^b^ (df = 1) | .69 (.01) | .58 (.02) | .88 (.001) | .92 (<.001) |

Cells show the *p*-values and the corresponding effect size computed as η_p_^2^ in brackets. Asterisk (*) indicates statistically significant effect at *p* < .05. Total df for all models = 41. Model 1 included the covariates age, sex, and education level; model 2 added diet and exercise level; model 3 additionally included BMI and clinical conditions (hypertension, sleep disorder, hyperlipidemia, diabetes mellitus, heart diseases); model 4 added sleep medication, anti-inflammatory medication, and antibiotics intakes

^a^ Participants were divided into two actigraphy-derived clusters based on spectral clustering

^b^ Participants were divided into two PSQI-based groups according to a total score cut-off of 5


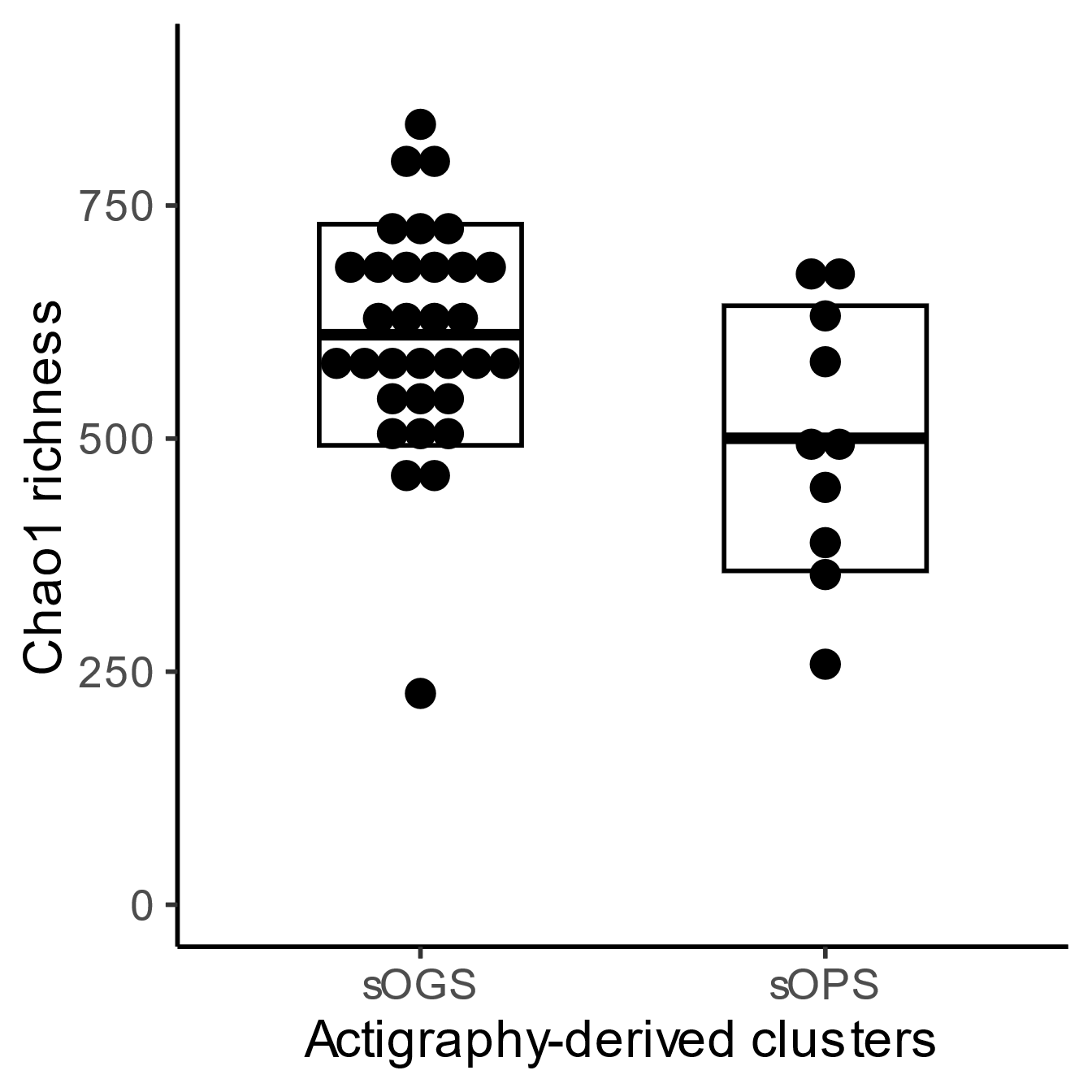


**Supplementary Fig. 2** Significant difference in Chao1 richness according to actigraphy-derived objective sleep measures. Participants were divided into two clusters based on spectral clustering. Dots represent individual data points. Crossbars represent group means ± standard deviation. The difference was statistically significant at p < .05. sOGS = Objective Good Sleepers based on spectral clustering, sOPS = Objective Poor Sleepers based on spectral clustering

**Supplementary Table 3** Permutation-based ANOVAs testing the associations between sleep and gut microbiota composition at phylum level

|  | **Actigraphy**^a^ | **PSQI**^b^ |
| --- | --- | --- |
| *Firmicutes* | .18 (.06) | .67 (.002) |
| *Bacteroidetes* | .02* (.13) | 1.00 (<.001) |
| *Proteobacteria* | .32 (.03) | .80 (.002) |
| *Actinobacteria* | .30 (.01) | .86 (.03) |

Cells show the *p*-values and the corresponding effect size computed as η_p_^2^ in brackets. Asterisk (*) indicates statistically significant effect at *p* < .05. No effect survived the multiple comparison correction (*p* < .01). Total df for each test = 41. The model included the covariates age, sex, and education level

^a^ Participants were divided into two actigraphy-derived clusters based on k-means clustering

^b^ Participants were divided into two PSQI-based groups according to a total score cut-off of 5

**Supplementary Table 4** Permutation-based ANOVAs testing the associations between sleep and gut microbiota composition at genus level

|  | **Actigraphy**^a^ | **PSQI**^b^ |
| --- | --- | --- |
| *Prevotella* | 0.88 (0.003) | 0.94 (0.002) |
| *Bacteroides* | 0.07 (0.10) | 0.22 (0.03) |
| *Clostridium XlVa* | 0.24 (0.03) | 1.00 (<.001) |
| *Parabacteroides* | 0.94 (<.001) | 0.10 (0.08) |
| *Lachnospiracea incertae sedis* | 0.98 (0.003) | 0.46 (0.04) |
| *Alistipes* | 0.77 (0.01) | 0.27 (0.03) |
| *Ruminococcus* | 0.02* (0.12) | 0.34 (0.02) |
| *Oscillibacter* | 0.66 (0.04) | 0.64 (0.01) |
| *Butyricimonas* | 0.94 (0.001) | 0.12 (0.10) |
| *Veillonella* | 0.004* (0.19) | 0.92 (0.02) |
| *Blautia* | 0.96 (0.003) | 1.00 (0.001) |
| *Eubacterium* | 0.12 (0.08) | 0.82 (0.004) |
| *Clostridium IV* | 0.96 (<.001) | 0.96 (0.003) |
| *Phascolarctobacterium* | 1.00 (<.001) | 0.52 (0.02) |
| *Faecalibacterium* | 0.35 (0.01) | 0.14 (0.07) |
| *Odoribacter* | 0.50 (0.06) | 0.82 (0.03) |
| *Clostridium XlVb* | 0.77 (0.01) | 0.86 (0.001) |
| *Roseburia* | 0.49 (0.01) | 0.77 (0.05) |
| *Citrobacter* | 0.98 (0.004) | 1.00 (<.001) |
| *Parasutterella* | 0.58 (0.04) | 1.00 (0.004) |
| *Ruminococcus2* | 1.00 (<.001) | 0.84 (0.01) |
| *Streptococcus* | 1.00 (<.001) | 0.30 (0.02) |
| *Coprococcus* | 0.08 (0.08) | 0.20 (0.04) |
| *Anaerostipes* | 0.63 (0.02) | 0.53 (0.01) |
| *Bifidobacterium* | 0.26 (0.02) | 0.98 (0.02) |
| *Clostridium XVIII* | 0.80 (0.03) | 0.36 (0.02) |
| *Flavonifractor* | 0.34 (0.02) | 0.32 (0.03) |
| *Intestinimonas* | 0.52 (0.02) | 1.00 (0.002) |
| *Collinsella* | 0.63 (0.01) | 0.03* (0.14) |
| *Gemmiger* | 0.82 (0.002) | 0.92 (<.001) |
| *Haemophilus* | 0.63 (0.02) | 0.86 (0.01) |
| *Erysipelotrichaceae incertae sedis* | 0.09 (0.08) | 1.00 (0.001) |
| *Dorea* | 0.84 (0.004) | 0.55 (0.003) |
| *Bilophila* | 0.56 (0.01) | 0.57 (0.01) |
| *Pseudoflavonifractor* | 0.34 (0.03) | 0.94 (<.001) |
| *Anaerotruncus* | 0.20 (0.03) | 0.62 (0.002) |
| *Escherichia Shigella* | 0.73 (0.003) | 0.71 (0.003) |
| *Enterobacter* | 0.51 (0.02) | 0.48 (0.01) |
| *Holdemania* | 0.84 (0.01) | 0.04* (0.09) |
| *Romboutsia* | 0.19 (0.03) | 0.53 (0.02) |
| *Fusicatenibacter* | 0.67 (0.01) | 0.86 (0.02) |
| *Granulicatella* | 1.00 (0.002) | 0.52 (0.03) |

Cells show the *p*-values and the corresponding effect size computed as η_p_^2^ in brackets. Asterisk (*) indicates statistically significant effect at *p* < .05. No effect survived the multiple comparison correction (*p* < .001). Total df for each test = 41. The model included the covariates age, sex, and education level

^a^ Participants were divided into two actigraphy-derived clusters based on k-means clustering

^b^ Participants were divided into two PSQI-based groups according to a total score cut-off of 5

**Supplementary Table 5** Permutation-based ANOVAs testing the associations between cognitive performance and emotional well-being with objective sleep quality

|  | **Total df** | **Actigraphy**^a^ |
| --- | --- | --- |
| HAM-A | 87 | .96 (<.001) |
| HAM-D | 87 | .61 (.001) |
| CERAD-NB Memory Total | 86 | .05 (.05) |
| ECog | 65 | 1.00 (<.001) |

Cells show the *p*-values and the corresponding effect size computed as η_p_^2^ in brackets. No significant effect was found at *p* < .05. Bonferroni-corrected threshold for significance was set at *p* < .01. The model included the covariates age, sex, and education level

^a^ Participants were divided into two actigraphy-derived clusters based on k-means clustering

**Supplementary Table 6** Permutation-based ANOVAs testing the associations between cognitive performance and emotional well-being with gut microbiota richness

|  | **Total df** | **Chao1 index** |
| --- | --- | --- |
| HAM-A | 37 | 1.00 (.004) |
| HAM-D | 37 | 1.00 (.002) |
| CERAD-NB Memory Total | 36 | .49 (.02) |
| ECog | 15 | .34 (.08) |

Cells show the *p*-values and the corresponding effect size computed as η_p_^2^ in brackets. No significant effect was found at *p* < .05. Bonferroni-corrected threshold for significance was set at *p* < .01. The model included the covariates age, sex, and education level
